# Contact Tracing in Healthcare Settings During the COVID-19 Pandemic Using Bluetooth Low Energy and Artificial Intelligence—A Viewpoint

**DOI:** 10.3389/frai.2021.666599

**Published:** 2021-04-23

**Authors:** Guanglin Tang, Kenneth Westover, Steve Jiang

**Affiliations:** Medical Artificial Intelligence and Automation (MAIA) Laboratory, Department of Radiation Oncology, University of Texas Southwestern Medical Center, Dallas, TX, United States

**Keywords:** COVID-19, deep learning, artificial intelligence, real-time location system, bluetooth, contact tracing, healthcare

## Abstract

The COVID-19 pandemic has inflicted great damage with effects that will likely linger for a long time. This crisis has highlighted the importance of contact tracing in healthcare settings because hospitalized patients are among the high risk for complications and death. Moreover, effective contact tracing schemes are not yet available in healthcare settings. A good contact tracing technology in healthcare settings should be equipped with six features: promptness, simplicity, high precision, integration, minimized privacy concerns, and social fairness. One potential solution that addresses all of these elements leverages an indoor real-time location system based on Bluetooth Low Energy and artificial intelligence.

## Background

As of March, 2021, the COVID-19 pandemic has infected over 115 million people and led to more than 2.5 million deaths worldwide^1^. Presymptomatic and asymptomatic transmission via airborne respiratory droplets and aerosol allows COVID-19 to spread rapidly, leading to deaths and significant economic damage (Huff and Singh, 2020; Pollock and Lancaster, 2020).

Despite extensive measures to control the spread of COVID-19, many researchers believe that it will linger for many years (Kissler et al., 2020). There is no effective treatment for COVID-19 so far, and we may not have one in the near future. Although a few vaccines (Baden et al., 2020; Polack et al., 2020) have been developed recently and obtained emergency use authorization from U.S. Food and Drug Administration (FDA), their availability is currently limited and the deployment to the majority of people may take quite a few months or years. There are also many outstanding questions regarding when herd immunity through natural infection might occur, making it difficult to estimate the cost of such an approach. One prediction based on current death toll data estimates the cost to be ~2.5 deaths per thousand people to achieve herd immunity (Hernandez-Suarez et al., 2020). Emerging COVID variants also make it difficult to predict when COVID-19 will resolve. Worse yet, lack of compliance with social distancing and mask wearing contributed to the deadly winter outbreak of COVID-19 in the US. These factors together suggest that the COVID-19^1^ pandemic will remain a threat to the world in a long run and that the main methods of control will be timely testing and self-quarantining of infected individuals.

## Problem and Existing Solutions

Healthcare settings are a special concern for COVID-19 spread because these areas simultaneously house a high concentration of COVID-19 infected patients and other populations that are at high risk of death should they contract COVID-19. Although the transmission rate of COVID-19 in healthcare settings has not yet been fully characterized, current estimates suggest that 20% of patient infections and 89% of healthcare worker infections have occurred in hospitals (Evans et al., 2020). The U.S. estimates that healthcare workers constitute 5% of the its population but have accounted for 16% of its COVID-19 infections (CDC COVID-19 Response Team, 2020). Healthcare worker infections have a compounding effect because quarantining these workers decreases the healthcare system's capacity. The high rate of asymptomatic infections and the long period of viral shedding create difficult challenges for contact tracing, especially among healthcare providers (Long et al., 2020).

Current manual contact tracing approaches are problematic because of their low precision and long latency in reacting to exposures. In cases of suspected exposures, it takes time, if feasible at all, to contact potentially exposed patients and ask about their activities in healthcare facilities within the last 2 weeks. Moreover, memory is susceptible to failure given these parameters. Manual contact tracing also raises privacy concerns, as some patients may be unwilling to share personal information about their activities. The tracing process is also tedious and slow. Digital approaches based on underlying communications between smartphones via Bluetooth are more attractive than manual approaches because they are simpler, more precise, and more prompt than manual contact tracing. Such approaches can quickly inform people who may have been exposed with automatic messages. These can also provide instructions for follow-up testing and quarantining if a close contact tests positive. To address these problems, a number of smartphone apps have been deployed (Colizza et al., 2021; Rodríguez et al., 2021). However, these approaches have not translated well to healthcare settings and suffer from several drawbacks. In particular, despite efforts to protect privacy, privacy concerns remain regarding use of smartphones for contact tracing, preventing general deployment of this approach. For example, Singapore used such an approach for contact tracing, but it was met with a privacy backlash (Cho et al., 2020). Additionally, this approach relies on personal smartphones. This is problematic given that those at highest are typically older or lower income. Not only does this raise concerns for feasibility, it also raises concerns related to social inequality.

## Solution Framework

To effectively deal with these challenges, we propose that healthcare contact tracing approaches should have the following features: (1) *Promptness*. This aspect is critical for reducing the number of exposures originating from a specific infected patient or healthcare worker. Automation and a streamlined response process are essential to achieving promptness. (2) *Simplicity*. Effortless use and management of the system will reduce human errors and costs, and increase the willingness of patients and healthcare workers to adopt it. Key components of simplicity include well-developed user interfaces and unobstructed communication. (3) *High precision*. Common sense and our current understanding of the data suggest that distance and time are the two factors that influence the viral transmission of COVID-19. For an automated contact tracing solution to give actionable data, it must accurately measure these parameters for interactions between individuals. By their nature, these systems also have the potential to evaluate whether subjects have donned facial masks or shields, which may further refine estimates of transmission probability. (4) *Integrable*. A good contact tracing system should be integrated with electronic healthcare record systems. Healthcare systems are unique in this regard, since they will typically have access to such data. (5) *Private*. Privacy concerns can be major barriers to the adoption of contact tracing techniques, since they lower patients' willingness to get involved. A good contact tracing system in healthcare settings should protect patient or staff privacy and alleviate their concerns about privacy. Possible approaches may include limiting tracing activities to within the hospitals and making system-generated data available to users, without sacrificing privacy. (6) *Fair*. It is socially unfair to exclude some patients because they lack access to certain digital technologies. For example, many older and low-income patients may not have access to smartphones.

## Bluetooth Low Energy Technology and Artificial Intelligence

Although it will be challenging to develop and implement COVID-19 contact tracing systems for healthcare settings that include all of the above features, the current state of technology can provide elegant solutions. A real-time location system (RTLS) based on Bluetooth Low Energy (BLE) and artificial intelligence (Tang et al., 2020) is a good candidate. In this approach, sensors are installed in an array throughout the hospital to measure signals transmitted from small BLE tags worn by patients, visitors, and healthcare workers. With the assistance of a deep learning algorithm, this system can automatically determine interpersonal distance and duration of contacts with great accuracy. Specifically, the trained AI algorithm can compare signals measured by all sensors (so-called fingerprint) of two tags to determine their distance. Considering the temporal dependence of distance (e.g., distance cannot change suddenly because of limited speed), a recurrent neural network (RNN), specifically the Long Short-Term Memory (LSTM) network, which has proved effective in accurately localizing tags in RTLS, is capable of distance tracking. This system also has the potential for integration with the electronic healthcare record and hospital surveillance systems to gather additional information about patients' demographic and medical information and facial mask/shield wearing compliance to more precisely estimate the infection risk and potential severity of a contact. Concerns about privacy can be alleviated by limiting the technology's use to specific hospital locations and to certain individuals, namely patients, visitors, and healthcare workers. It can be offered to every patient and visitor, whether they own a smartphone or not. It can be highly recommended to all healthcare workers and visitors to maximize effectiveness. Such a technology could be a highly precise, prompt, simple, and socially fair approach to contact tracing in healthcare settings that would have minimal privacy concerns.

In addition to BLE, other technologies have been used for indoor RTLS such as passive RFID (Radio-frequency identification), UWB (Ultra-Wideband), IR (infrared radiation) and Ultrasound. While these technologies also have potential for contact tracing, their shortcomings may prevent their wide clinic applications. For example, UTB and Ultrasound has a low cost-efficiency, and passive RFID and IR has limited range. Comparing to these technologies, BLE is very cost-efficient and has basically no blind spots in clinic settings. On the other hand, BLE also has shortcoming, e.g., relatively low accuracy. However, this has been recently solved by increasing number of sensors and employing deep learning technologies. In the 3-storage radiation oncology building at University of Texas Southwestern Medical Center, a BLE sensor network consisting of 142 sensors each costing ~$50 with a deep learning algorithm based on LSTM achieved 100% of zone-localization accuracy. The total cost of such a system including hardware, construction, and software development was ~$10,000 for a 63,000-square-foot facility (Tang et al., 2020).

## Conclusion

The COVID-19 pandemic has continued for over a year, infecting and killing millions of people. Many individuals are still at risk, and reinfection is known to happen, so it is likely that the pandemic will linger. Effective contact tracing technologies are urgently needed for healthcare settings that contain a high concentration of older adults and people with underlying conditions that increase the risks of COVID-19 infection. However, existing or currently proposed contract tracking technologies suffer from major challenges such as privacy concerns and limited access to the economically disadvantaged. A good contact tracing technology for healthcare settings should satisfy six requirements: promptness, simplicity, high precision, integrable, private, and fair.

Based on the success of using BLE and deep learning for accurate localization by RTLS, this technology can readily be adapted to contact tracing in healthcare settings. Such a system would meet the six requirements we propose as part of a good contact tracing technology in healthcare settings. We believe that such technology will not only be useful in controlling COVID-19, it is generally applicable to other infectious diseases.

## Data Availability Statement

The original contributions presented in the study are included in the article/supplementary material, further inquiries can be directed to the corresponding author/s.

## Author Contributions

All authors listed have made a substantial, direct and intellectual contribution to the work, and approved it for publication.

## Conflict of Interest

The authors declare that the research was conducted in the absence of any commercial or financial relationships that could be construed as a potential conflict of interest.

## Footnotes

^1^*John Hopkins Corona Virus Resource Center*. Available online at: https://coronavirus.jhu.edu/map.html (accessed March 2, 2021).
